# What are the job attribute preferences of physicians and nurses in Türkiye? Evidence from a discrete choice experiment

**DOI:** 10.1186/s12960-023-00826-4

**Published:** 2023-06-28

**Authors:** Elif İşlek, Bayram Şahin

**Affiliations:** 1 Faculty of Health Sciences, Department of Social Work, Bartın University, Ağdacı mah, Merkez, 74100 Bartın, Türkiye; 2 Faculty of Economics and Administrative Sciences, Department of Healthcare Management, Hacettepe University, Beytepe, Çankaya, 06800 Ankara, Türkiye

**Keywords:** Human resources for health, Türkiye, Discrete choice experiment, Nurse, Physician, Incentive, Job preference, Willingness to Pay

## Abstract

**Background:**

In Türkiye, as in other countries, the maldistribution of the health workforce is a serious concern. Although policymakers have developed various incentive packages, this problem has not been thoroughly addressed yet. Discrete choice experiment (DCE) is a valuable method to provide evidence-based information for these incentive packages to attract healthcare staff for rural jobs. The main aim of this study is to investigate the stated preferences of physicians and nurses when choosing a job region.

**Methods:**

A labelled DCE was conducted to assess job preferences of physicians and nurses from two hospitals one of which is urban, and the other is in a rural region in Türkiye Job attributes included wage, creche, infrastructure, workload, education opportunity, housing, and career opportunity. Mixed logit model was used to analyse the data.

**Results:**

The strongest attribute associated with job preferences was region (coefficient − 3.06, [SE 0.18]) for physicians (*n* = 126) and wages (coefficient 1.02, [SE 0.08]) for nurses (*n* = 218). According to the Willingness to Pay (WTP) calculations, while the physicians claimed 8627 TRY (1,813 $), the nurses claimed 1407 TRY (296 $) in addition to their monthly salaries to accept a rural job.

**Conclusion:**

Both financial and non-financial factors did affect the preferences of physicians and nurses. These DCE results provide information for policymakers about what characteristics might increase the motivation of physicians and nurses to work in rural areas in Türkiye.

## Introduction

The planning and management of human resources are quite challenging due to the considerable time and effort involved in training physicians and nurses, establishing medical schools, and implementing policies for improving human resources in the healthcare field. [1]. Therefore, one of the most important challenges that both developed and developing countries face is the scarcity of healthcare workers in rural regions. As such, many studies emphasize maldistribution of health workers as an crucial health system issue [1–7].

Health professionals tend to work in economically, physically, and socially attractive areas rather than rural and remote areas [3, 4]. A poor workforce distribution is a significant obstacle to ensuring equity in the provision of health services and reducing interregional disparities in health indicators [2]. Several countries have been looking for a solution to decrease regional disparities and provide equity in access to high-quality healthcare services for many years. Providing a higher wage, using educational incentives (e.g., opening health schools in rural areas) and educational financial incentives (e.g., providing scholarships or loans for graduates to undertake a certain period of service in rural areas), limiting work (e.g., contract-based jobs) in urban areas where there is a greater number of physicians, and providing additional payments have all been successful in attracting people to work in rural areas [2–8].

Many reforms and policy initiatives concerning human resources in the healthcare system have been attempted since the Republic of Türkiye’s foundation. Although the implementation of a series of reforms such as compulsory public service for physicians after their graduation, introducing restrictions of personnel relocations using distribution scales in personnel assignment, contracting personnel in rural areas, and introducing a performance-based payment system have made significant contributions on many issues ranging from increasing the number of personnel to mitigating distribution inequalities, the problems stemming from the shortage of healthcare personnel persist. National statistics in Türkiye confirm the shortage of healthcare personnel and unequal distribution. According to national statistics from 2018, the number of physicians and nurses in patient care per 100,000 resident population is 187 and 301, respectively. This is substantially lower than the European Union average of 371 physicians and 841 nurses per 100,000 resident population, as well as the OECD average of 348 physicians and 938 nurses. Furthermore, while physician density in Türkiye exceeds 188 in the best-performing provinces, it falls below 130 in the worst-performing ones. For nurses, it exceeds 350 in the best-performing provinces but falls below 272 in the worst-performing ones [9].

While developing incentive packages for healthcare workers to voluntarily work in rural or underserved areas is a more challenging task for policymakers than mandating compulsory service, the literature emphasizes that healthcare workers who volunteer to work in rural or resource-limited areas stay longer than contract and compulsory service workers [10]. Thus, employee satisfaction and willingness to stay in a region are critical factors in achieving the targets of healthcare reforms.

According to the last national survey in Türkiye on healthcare worker satisfaction, 64.6% of workers were generally satisfied with their jobs, while 13.5% are dissatisfied [11]. In a study of workers from several industries conducted by Sagir et al. healthcare workers were found to be the least motivated [12].

Since the onset of the COVID-19 pandemic in early 2020, all healthcare workers, particularly physicians and nurses, have faced increased workload and burnout. As such, an estimated 333,942 healthcare providers left their jobs in 2021, many for pandemic-related causes such as burnout, long hours, heavy patient loads, and personal health concerns [13]. Given that health care quit rates are a growing concern for public health systems, an accurate assessment of the obstacles to healthcare staff motivation is critical. Unveiling the obstacles could provide evidence for policymakers to put a greater emphasis on incentives that will increase the motivation of healthcare workers. However, there is insufficient evidence, including quantitative findings, on what strategies improve healthcare workforce performance, how wage or working conditions affect motivation and choice, what defines work choices, the importance of factors influencing work choice, and how much compromise is made on other factors [14–19].

The discrete choice experiment (DCE) is one of the methods used to study the choices of individuals. DCE has been widely used in the field of healthcare economics in recent years [20–22]. It is a relatively affordable and valuable method that can provide information to decision-makers when determining strategies to address healthcare workforce problems. Although DCE is mostly used to assess consumer choices in healthcare services, it is also used to determine job and employment alternatives for service providers [15, 16, 18, 19, 24–29]. It provides quantitative information on issues such as which work attributes and working conditions are regarded as important by healthcare workers, which attribute is regarded as more important than another, how much wage will be sacrificed for improvements to various work attributes, and what the level of probability of accepting a job with certain attributes is [8].

Considering the ongoing unequal distribution of the healthcare workforce across regions in Türkiye, very few studies have reported quantitative results on the preferred job attributes of healthcare workers. This study attempted to address this critical gap by examining the relative importance of key job attributes on physicians' and nurses' job preferences, revealing how much they were willing to sacrifice from their salary to have certain attributes and providing recommendations for incentive packages to policymakers by calculating the acceptance probability of incentive packages developed with specific job attributes.

## Materials and methods

### Survey sample

The study was conducted in two state hospitals in two different provinces selected at random from the 1st and 6th regions according to the grouping of the service regions of the Ministry of Health in respect of the differences that can be seen between urban and rural regions in Türkiye. The first region, comprising eight provinces, has been deemed to possess the most favourable socio-economic status since 2017, with Ankara being chosen at random as the urban area within this region. As for the sixth region, which comprises 17 provinces, Ağrı was randomly selected as the rural area. In Ağrı, the Ağrı State Hospital was selected as the only large hospital, and in Ankara, the Ankara Gazi Mustafa Kemal State Hospital was selected as the closest in bed capacity and institutional type to the hospital in Ağrı. It was attempted to reach all the physicians and nurses actively working in each hospital without sampling.

### Discrete choice experimental design

This questionnaire-based, cross-sectional DCE study was conducted based on the accumulated knowledge on DCE methodology extensively utilized in the literature [8, 16, 19, 26, 28, 30–41]. Qualitative research was conducted to determine the most important attributes that affect the choice of job and working conditions of physicians and nurses to determine the variables, which is the first step of DCE. A semi-structured interview form was prepared from a scan of national and international literature, which was first used in focus group interviews and then in detailed interviews. The focus group with 8 nurses and in-depth interviews with 6 physicians were conducted in Ankara Ulus State Hospital. The most important variables affecting the job preferences of nurses and physicians were determined according to the results of qualitative research (Table 1).

**Table 1:** Job attributes and sub-levels of physicians and nurses

Job attributes	Levels	
	Physician	Nurse
1. Wage	1. 6000–10,999 TRY	1. 3000–4499 TRY
	2. 11,000–15,999 TRY	2. 4500–5999 TRY
	3. 16,000–21,000 TRY	3. 6000–7500 TRY
2. Creche	1. Not available	1. Not available
	2. Available	2. Available
3. Infrastructure	1. Insufficient	1. Insufficient
	2. Sufficient	2. Sufficient
4. Education opportunity	1. Not available	1. Not available
	2. Available	2. Available
5. Housing	1. Not available	1. Not available
	2. Available	2. Available
6. Workload	1. More than 40 h a week	1. More than 40 h a week
	2. Up to 40 h a week	2. Up to 40 h a week
7. Career opportunity	1. Not available	1. Not available
	2. Available	2. Available

*TRY* Turkish Lira

**Fig. 1:**
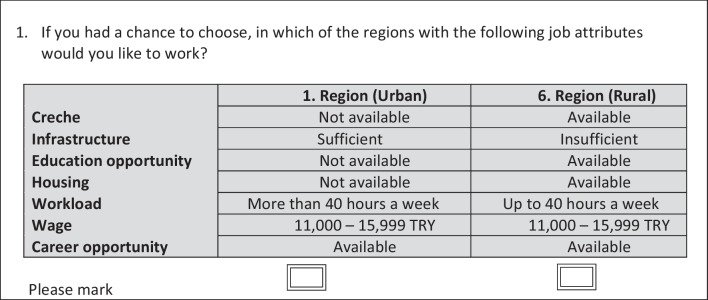
Example of choice set (translated into English from the original Turkish version)

These variables were used within the experimental design. From the 7 variables and their sub-levels with full factorial design according to the formula n(n-1)/2, a unique hypothetical choice set can be formed; *n* = 2^6^ × 3^1^ = 192 scenario/alternative and 192 × (192–1)/2 = 18,336 choice sets would be generated, and those were not feasible for a single individual to choose. Accordingly, 36 choice sets were constructed by using a D-efficient design with Ngene DCE design software. In order to lessen the burden of participants, Ngene software was also employed to divide 36 choice sets into two versions with the block method in the creation of the design. Thus, each participant was questioned about 18 pairs of scenarios. The questionnaires were created separately for physicians and nurses and were prepared. An example of the choice sets is demonstrated in Fig. 1.

### Data collection

The pilot study was conducted with 20 participants (10 physicians, 10 nurses) in Ankara Ulus State Hospital in February 2018. Evaluations were made in respect of whether or not the questionnaire was valid, comprehensibility, and how many minutes it took to complete the form. As a result of the pilot study, the sociodemographic questions were corrected, changing the labels of 1st and 6th region to Ankara and Ağrı (as it was seen that some subjects had experience specific to the province and this affected their choice of attributes), the explanations of the sub-level variables were made more comprehensible, and it was decided that it would be more effective to make the field application face-to-face.

The study was conducted in Ankara Gazi Mustafa Kemal State Hospital (1st region) and in Ağrı State Hospital (6th region) between October 2018 and January 2019. All the actively working physicians and nurses were included and sampling was not calculated. In the two hospitals where the research was conducted, there were 220 physicians and 376 nurses employed. After the identification of those not actively working because of temporary duty, sick leave, holiday leave, etc., 466 personnel, comprising 165 physicians and 301 nurses were determined to be actively working. Finally, 126 physicians and 218 nurses participated in the study, which gave a response rate of voluntary participation of 74%.

The two groups of questionnaires were distributed equally to the physicians and nurses working in each unit of the hospital taking age and gender into consideration [42]. The aim of the research and method were explained to each participant to ensure that these had the same meaning in the scenarios for each participant.

### Data analysis

The mixed logit regression model has been widely used in DCE data analysis, because this method allows heterogeneity in the preferences between participants and allows unrelated alternatives independent of the assumption [34, 39, 43, 44]. In the modelling of preferences repeated by the same person, the mixed logit model has been reported to provide better model fit than other logistic models [33, 34]. The research model performed in the Stata statistical program mixed logit regression analyses was as shown below:

U_nit_ = (*β*_1_ + *ƞ*_*1n*_) region + (*β*_2_ + *ƞ*_*2n*_) wage + (*β*_3_ + *ƞ*_*3n*_) creche_available + (*β*_4_ + *ƞ*_*4n*_) infrastructure_sufficient + (*β*_5_ + *ƞ*_*5n*_) education_available + (*β*_6_ + *ƞ*_*6n*_) housing_available + (*β*_7_ + *ƞ*_*7n*_) workload_normal + (*β*_8_ + *ƞ*_*8n*_) career opportunities_available + *Ɛ*_nit_.

As the research design was a labelled model, the alternative attribute (region) was added to the model and beta coefficients were interpreted as for the other variables [45], because region was effective in the preferences of the respondents and the benefit between the alternatives was affected by the value. According to the results of the mixed logit analysis, beta coefficients were interpreted as scale conversions of the marginal benefit of each attribute [26, 46]. In other words, the regression coefficients stated the relative importance of the attributes numerically [47]. In addition to the effects on the working condition attributes, the possibilities of the physicians and nurses accepting a job with the wage desired and the attributes determined were calculated.

Willingness to pay (WTP) is a measure of how much participants value certain attributes. WTP is calculated by comparing of the coefficients of attribute levels to monthly income. Positive results indicate how much participants are willing to pay to pay/sacrifice in order to have a trait level [25], whereas negative results indicate the minimum amount participants are willing to be compensated for a particular attribute level [26]. A simulation study is also conducted on how policy interventions, such as changing job attributes, can affect the uptake rates of nurses and physicians for rural vs. urban jobs.

## Results

### Demographic findings

The study included 126 physicians with a mean age of 37 years and 218 nurses with a mean age of 33 years. The majority of physicians were male and the majority of nurses were female. The demographic characteristics of the study participants are shown in Table 2.

**Table 2:** Demographic characteristics of the participants

	Physician		Nurse	
	*n*	%	*n*	%
Age				
30 years and under	33	26.2	95	43.6
31–40 years	60	47.6	76	34.8
41 years and older	33	26.2	47	21.6
Gender				
Female	55	43.7	175	80.3
Male	71	56.3	43	19.7
Marital status				
Single	47	37.3	70	32.1
Married	79	62.7	148	67.9
Speciality				
GP	25	19.8	–	–
Specialist	101	80.2	–	–
Education				
High school	–	–	15	6.9
Two-year degree	–	–	48	22.0
Graduate	–	–	144	66.1
Postgraduate	–	–	11	5.0
Work experience				
1–10 years	77	61.1	112	51.4
11–20 years	27	21.4	70	32.1
21–30 years	13	10.3	34	15.6
31 years and above	9	7.2	2	0.9
Rural work experience				
No	48	38.1	133	61.0
Yes	78	61.9	85	39.0

### Findings of the preferences of job attributes of the physicians and the nurses

When stating preferences related to working conditions, the physicians gave most importance to the region compared to other variables with a β coefficient of -3.06. There was a lower probability of physicians choosing to work in a rural region than in an urban region. The importance of working condition attributes after region were seen to be wage, education opportunities, workplace infrastructure, creche availability, career opportunities, and workload, respectively. The variable of the provision of accommodation, as another working condition attribute in the model, was not seen to be statistically significant in the choice of region of work for physicians (*p* = 0.701).

When the nurses were stating preferences related to working conditions, the most importance was given to wage compared to other variables with a β coefficient of 1.01, followed by region with a β coefficient of -0.95. There was a lower probability of nurses choosing to work in a rural region than in an urban region. The importance given to subsequent working condition attributes was workplace infrastructure, workload, education opportunities, provision of accommodation, career opportunities, and creche availability, respectively (Table 3).

**Table 3:** The mixed logit analysis results related to the job attributes of the physicians and nurses

Preference	Coefficient *β*	SE	z	*p*	CI (95%)	
**Physician **(*n* = 128)						
Rural region	− 3.06	0.18	− 17.18	< 0.001	(− 3.41, − 2.71)	
Wage	1.83	0.21	8.91	< 0.001	(1.43, 2.23)	
Creche	0.62	0.16	3.86	< 0.001	(0.30, 0.93)	
Infrastructure	0.65	0.18	3.56	< 0.001	(0.29, 1.01)	
Education opportunity	0.86	0.22	3.87	< 0.001	(0.42, 1.29)	
Housing	− 0.05	0.13	− 0.38	0.701	(− 0.30, 0.20)	
Workload	0.58	0.18	3.19	< 0.001	(0.22, 0.93)	
Career opportunity	0.60	0.13	4.72	< 0.001	(0.35, 0.84)	
**Nurse** (*n* = 216)						
Rural region	− 0.96	0.05	− 20.57	< 0.001	(− 1.05, − 0.87)	
Wage	1.02	0.08	12.94	< 0.001	(0.86, 1.17)	
Creche	0.23	0.06	3.59	< 0.001	(0.10, 0.35)	
Infrastructure	0.38	0.07	5.64	< 0.001	(0.25, 0.52)	
Education opportunity	0.27	0.09	3.17	< 0.002	(0.10, 0.43)	
Housing	0.25	0.06	4.08	< 0.001	(0.13, 0.37)	
Workload	0.36	0.08	4.61	< 0.001	(0.21, 0.52)	
Career opportunity	0.24	0.06	3.91	< 0.001	(0.12, 0.36)	

*p* < 0.05

### The findings related to the job attribute of WTP of physicians and nurses

According to the Willingness to Pay (WTP) calculations of the physicians, more than 8,627 TRY (1,813 $) was desired to work in the 6th region, and there was WTP 2,563 TRY (539 $) from the wage if there was an educational opportunity for themselves, 1,963 TRY (413 $) for infrastructure, 1,729 TRY (363 $) for career opportunities, 1,694 TRY (356 $) for creche availability, and 1,445 TRY (304 $) for a normal workload. As the variable of provision of housing was not found to be statistically significant (*p* = 0.70), it was not included in the WTP calculations for physicians.

According to the WTP calculations for the nurses, more than 1,407 TRY (296 $) was desired to work in the 6th region, and there was WTP 394 TRY (83 $) from the wage if there was an educational opportunity for themselves, 566 TRY(119 $) for infrastructure, 349 TRY(73 $) for career opportunities, 332 TRY(70 $) for creche availability, 368 TRY(77 $) for provision of housing, and 533 TRY (112 $) for a normal workload (Table 4).

**Table 4:** WTP related to the alternative working conditions of the physicians and nurses

Job attributes	Levels	Physician		Nurse	
		Coefficient	WTP	Coefficient	WTP
Wage	1. Basic level	0.0003499	1	0.00068	1
	2. Second level				
	3. Third level				
Region	1. First. Region				
	2. Sixth Region	− 3.018914	− 8627 TRY	− 0.95882	− 1407 TRY
Creche	1. No				
	2. Yes	0.593079	1694 TRY	0.22655	332 TRY
Infrastructure	1.Insufficient				
	2. Sufficient	0.687138	1963 TRY	0.38568	566 TRY
Education opportunity	1. No				
	2. Yes	0.8968191	2563 TRY	0.26900	394 TRY
Housing	1. No				
	2. Yes	–	–	0.25079	368 TRY
Workload	1. More than 40 h a week				
	2. Up to 40 h a week	0.5057698	1445 TRY	0.36346	533 TRY
Career opportunity	1. No				
	2. Yes	0.6051909	1729 TRY	0.23819	349 TRY

### Predicted probabilities of the job preferences of the physicians and nurses

The working condition attributes of different incentive packages prepared by policy-makers were observed to change the probability of the nurses choosing to work in a rural region. When all other variables were fixed, an increase in wage from 3750 to 5250 TRY increased the probability of working in a rural region from 56 to 78%. Without any other incentive components, wage alone would have to be at least 14,000 TRY for a 100% probability of choosing to work in the 6th region. When other attributes were not met and the wage alone was 3750 TRY, the probability of accepting the package was 37% for the 1st region and 18% for the 6th region. With the addition of educational opportunities to this package, the probability of acceptance for the 1st and 6th regions, respectively, increased by 6% and 5%, with the addition of creche availability by 5% and 4%, with sufficient infrastructure by 9% and 7%, with a normal workload by 9% and 6%, with the provision of accommodation by 5% and 4%, and with career opportunities by 5% and 4%.

For physicians, when all other variables were fixed, an increase in wage from 8500 TRY to 13,500 TRY increased the probability of working in a rural region from 50 to 85%. Without any other incentive components, wage alone would have to be at least 34,000 TRY for 100% probability of choosing to work in the 6th region. When other attributes were not met and the wage alone was 8500 TRY, the probability of accepting the package was 43% for the 1st region and 4% for the 6th region. With the addition of educational opportunities to this package, the probability of acceptance for the 1st and 6th regions, respectively, increased by 22% and 4%, with the addition of creche availability by 15% and 2%, with sufficient infrastructure by 17% and 3%, with a normal workload by 13% and 2%, and with career opportunities by 15% and 2%.

## Discussion

Despite the numerous DCE-based studies conducted in the healthcare sector, most of them have been carried out in low-income countries. However, no such study has yet explored the preferences of healthcare workers in Türkiye, a middle-income country, regarding job roles and working conditions.

As in some previous studies [23, 34, 39, 48–52], the results of the current study showed that a high wage, educational opportunities, sufficient workplace infrastructure, creche availability, career opportunities, the provision of accommodation, and a normal workload had a positive effect on the preferences of physicians and nurses, whereas a rural region had a negative effect.

In contrast to these results, in a study of medical faculty students in Ghana, Kruk et al. found that the most important attributes in job preferences were provision of accommodation and sufficient infrastructure, and the least important attribute was wage [34]. These findings show the concerns of physicians in Ghana that in rural regions there will be accommodation problems and that healthcare institutions will not have sufficient infrastructure to be able to practice the profession. This constitutes proof of the need to match policies and incentive packages with the preferences and expectations of healthcare personnel [52] and to apply completely different combinations of human resource policies in different countries [42]. Thus, as the features of the healthcare system, the culture, expectations, and needs are different, the preferences of healthcare workers related to work and working conditions can show variability in studies conducted in different countries and regions. Furthermore, the significance of housing differs between low-income nurses and high-income physicians since the former have to allocate a larger percentage of their income to accommodation, despite the amount being the same in lira wise.

In this study, the most important attribute in the preferences of the physicians was the region, whereas for the nurses it was wage. Similarly, Rao et al. reported that the probability of nurses accepting a job in a rural region was higher than that of physicians [53]. It has been emphasized in the literature that there may be dominant attributes when individuals are selecting preferences [54]. It is thought that compared to nurses, as physicians have concerns about earning relatively more and not developing professionally, more importance is given to the region variable.

According to the mixed logit model results, region was identified as the most important attribute affecting the preferences of physicians across all subgroups, except those studying at universities in rural areas, who instead prioritized wages as the most important attribute. Similarly, region was also deemed the most important attribute for nurses in various subgroups, including those from the 1st region, female, over 41 years old, born in urban areas, studying at university in urban areas, married, with children, earning higher or lower incomes, newly hired, possessing extensive work experience, not on a contract, and suffering from chronic diseases (Annex).

The results of the study align with previous research, indicating that nurses living in urban areas expressed a strong preference to work in similar settings [3]. Participants' tendency to choose the status quo attribute may be explained by the “prospect theory”, which suggests that individuals making choices under uncertain conditions may deviate from the expected utility theory by prioritizing loss minimization over gain maximization [55]. As per Liu et al. study, individuals from urban areas and those with higher family incomes exhibit a lower preference for working in rural areas [49]. The findings of DCE studies conducted in Liberia and Vietnam suggest that healthcare workers born in rural areas are more willing to work in such regions [39, 56]. Furthermore, previous literature suggests that healthcare professionals studying at universities in rural areas are more likely to work in those areas [4, 7]. These results suggest that individuals who were born, raised, or educated in rural areas are less biased and more motivated to work in those regions than those with no such background. However, those who prioritize location over wages may prefer urban areas that offer access to tertiary health services, quality education for their children, and social activities.

The results of this study showed that both financial and non-financial attributes are important in the preferences of physicians and nurses related to working conditions. However, the working condition attributes were seen to have different levels of importance for physicians and nurses. The provision of accommodation was not found to be statistically significant in the preferences of the physicians, whereas for nurses it was more important than career opportunities and creche availability. Physicians gave more importance to career opportunities and nurses to educational opportunities.

According to the results of this study, the two most important attributes to which physicians and nurses paid attention when selecting preferences related to working conditions were the region itself and wage. The importance of wage and region was also supported by interviews and focus group discussion. Therefore, the wage will be the most motivating component that can be provided by policy-makers for physicians and nurses to accept a certain job. Changes to be made to salaries are both effective and provide a rapid result. However, an increase in wage alone may not be a sufficient intervention for physicians and nurses to accept a certain job, and this is not a method that can be implemented as policy in countries with limited resources, such as Türkiye. Based on the predicted probabilities of physicians' and nurses' job preferences, a threefold increase in wages for nurses and a fourfold increase for physicians would be necessary to achieve a 100% probability of selecting rural areas. In addition, as reported in the literature, the probability of it being sufficient and successful in the long term is low. However, effective interventions may be possible with the combination of incentive packages and different policies matched with the preferences and expectations of healthcare workers [3, 25, 43, 51]. Policy-makers should focus on other motivating components after deducting a certain level of monthly income [49]. This study's results suggest that the other attributes, aside from wage, had a relatively smaller impact on the probability of selecting a job in a rural area. Therefore, a combination of different attributes in incentive packages may be more effective in motivating healthcare workers to work in rural areas. These results will shed light on policy makers on the key factors driving the attractiveness and retention of physicians and nurses in rural areas.

There were a few limitations to this study. Firstly, it was conducted in two different secondary level hospitals located in two regions with varying levels of development. Therefore, it may not be possible to apply the results of the study to the entirety of Türkiye. Secondly, similar to other DCE studies, only the stated preferences of the participants were examined. It is uncertain whether these outcomes align with the preferences revealed through actual behaviour. Thirdly, the sample size was insufficient to carry out certain subgroup analyses. Lastly, the calculations for wages, USD exchange rates, and probabilities were based on average salaries and an exchange rate of 4.75 USD at the time of the research.

## Conclusion

To sum up, this DCE study provides strong evidence to policymakers about the job preferences of healthcare workers regarding working conditions in Türkiye. The results emphasized that both financial and non-financial factors affected the choices of physicians and nurses. The outcomes of this study also provide valuable insights for policymakers to create more attractive incentive packages for healthcare workers. Furthermore, incentive packages that meet the needs and expectations of physicians and nurses may encourage them to work with high motivation, decrease the turnover rate, and increase the retention rate in rural regions. This study can serve as a useful guide for further research, which should include other healthcare professionals working in primary and tertiary level healthcare institutions, including the private sector and different regions with a larger sample size.

## Data Availability

Available upon request.

## Annex

### Ranking of importance of job attributes according to subgroup analyses of physician

**Table:**

		Region	Wage	Creche	Infrastructure	Education	Housing	Workload	Career
**Physicians**	Average	1	2	5	4	3	0	7	6
Province	Ankara	1	2	6	3	4	0	0	5
	Ağrı	1	2	4	0	3	0	6	5
Sex	Female	1	2	5	7	3	0	4	6
	Male	1	2	6	4	3	0	0	5
Age	35 years and below	1	2	4	7	3	0	6	5
	36–49 years	1	2	0	4	3	0	0	5
	50 years and above	1	2	0	0	0	0	0	0
Hometown	1st region	1	2	0	5	3	0	4	6
	2nd 3rd and 4th region	1	2	6	3	5	0	4	7
	5th and 6th region	1	2	0	0	3	0	0	4
Hometown	Urban	1	2	4	6	3	0	7	5
	Rural	1	2	0	4	3	0	0	5
University location	1st and 2nd region	1	2	6	3	4	0	5	7
	3rd and 4th region	1	2	0	0	0	0	0	3
	5th and 6th region	2	1	4	0	3	0	0	0
Speciality	General practitioner	1	2	0	0	0	0	0	0
	Specialist	1	2	6	4	3	0	7	5
Marital status	Single	1	2	0	5	4	0	3	6
	Married	1	2	4	5	3	0	0	6
Having children	No	1	2	0	5	4	0	3	6
	Yes	1	2	4	6	3	0	0	5
Having a house	Yes	1	2	5	0	3	0	6	4
	No	1	2	5	4	3	0	6	7
Monthly income	8500 TRY and below	1	2	6	5	4	0	0	3
	8501 TRY and above	1	2	4	5	3	0	0	6
Year in working city	1 year	1	2	4	0	3	0	0	5
	2–9 years	1	2	5	3	4	0	6	7
	10 years and above	1	2	0	0	0	0	0	3
Number of worked places	1–3	1	2	7	3	4	0	5	6
	4 and above	1	2	5	0	3	0	0	4
Workload	40 h and below	1	2	0	0	0	0	0	0
	More than 40 h	1	2	6	5	3	0	7	4
Compulsory service	Completed	1	2	6	3	4	0	0	5
	Not completed	1	2	4	0	3	0	6	5
Rural experience	Yes	1	2	5	4	3	0	0	6
	No	1	2	6	0	3	0	5	4
Stated Health status	Bad and middle	1	2	0	0	0	0	3	0
	Good	1	2	6	4	3	0	0	5

0 = *p* > 0.05

### Ranking of importance of job attributes according to subgroup analyses of nurses

**Table:**

		Region	Wage	Creche	Infrastructure	Education	Housing	Workload	Career
**Nurses**	Average	2	1	8	3	5	6	4	7
Province	Ankara	1	2	0	5	4	6	3	7
	Ağrı	2	1	5	3	7	6	4	8
Sex	Female	1	2	8	4	6	7	3	5
	Male	2	1	6	3	0	5	4	0
Age	30 and below	2	1	7	5	4	0	6	3
	31–40	2	1	0	4	0	3	5	0
	41 and above	1	2	0	4	0	0	3	0
Hometown location	1st and 2nd region	1	2	6	5	0	3	0	4
	3rd and 4th region	1	2	0	0	3	4	0	5
	5th and 6th region	2	1	5	4	0	0	3	6
Hometown	Urban	1	1	8	4	7	6	3	5
	Rural	2	2	5	4	3	6	0	0
University location	1st and 2nd region	1	2	7	4	5	8	3	6
	3rd and 4th region	1	2	0	3	0	0	0	0
	5th and 6th region	3	1	6	4	0	5	2	7
Education	High school and assos. degr	2	1	5	4	0	3	0	0
	Bachelor deg. and above	2	1	8	4	5	7	3	6
Marital status	Single	2	1	0	4	0	0	5	3
	Married	1	2	7	4	5	6	3	0
Having children	No	2	1	0	4	6	0	5	3
	Yes	1	2	6	4	7	5	3	0
Having a house	Yes	2	1	6	4	0	7	3	5
	No	2	1	8	3	4	5	6	7
Monthly income	3750 TRY and below	1	2	6	4	5	7	3	8
	3751 TRY and above	2	1	8	3	5	7	4	6
Monthly household income	6000 TRY and below	1	2	0	3	0	4	0	0
	6001 TRY and above	1	2	4	6	5	8	3	7
Year in working city	1 year	1	2	7	4	5	0	3	6
	2–9 years	2	1	6	3	5	8	4	7
	10 years and above	1	2	0	3	0	4	5	0
Work experience	1 year	1	2	7	5	3	0	4	6
	2–9 years	2	1	5	3	6	7	4	8
	10 years and above	2	1	0	0	0	0	0	0
Number of worked places	1	2	1	7	3	6	0	4	5
	2 or 3	2	1	0	6	4	7	3	5
	4 and above	1	2	6	4	0	5	3	0
Workload	40 h and below	1	2	0	4	3	7	5	6
	More than 40 h	2	1	5	3	7	6	4	8
Having managerial duty	Yes	1	2	0	3	0	0	0	0
	No	2	1	8	3	5	6	4	7
Job type	Staff with cadre	1	2	7	4	6	5	3	8
	Contract-based	2	1	7	3	6	0	5	4
Rural experience	Yes	2	1	7	5	0	4	3	6
	No	2	1	7	3	5	8	4	6
Stated health status	Bad and middle	2	1	0	4	0	5	3	0
	Good	2	1	5	3	6	8	4	7
Having chronic disease	No	2	1	7	3	5	8	4	6
	Yes	1	2	0	0	0	3	4	0

0 = *p* > 0.05

## Abbreviation

DCE: Discrete choice experiment
WTP: Willingness to Pay
TRY: Turkish Lira
